# The Iranian version of 12-item Short Form Health Survey (SF-12): factor structure, internal consistency and construct validity

**DOI:** 10.1186/1471-2458-9-341

**Published:** 2009-09-16

**Authors:** Ali Montazeri, Mariam Vahdaninia, Sayed Javad Mousavi, Speideh Omidvari

**Affiliations:** 1 Department of Mental Health, Iranian Institute for Health Sciences Research, ACECR, Tehran, Iran; 2 Department of Social Medicine, Iranian Institute for Health Sciences Research, ACECR, Tehran, Iran; 3 Department of Physical Therapy, Faculty of Rehabilitation Sciences, Tehran University of Medical Sciences, Tehran, Iran

## Abstract

**Background:**

The 12-item Short Form Health Survey (SF-12) as a shorter alternative of the SF-36 is largely used in health outcomes surveys. The aim of this study was to validate the SF-12 in Iran.

**Methods:**

A random sample of the general population aged 15 years and over living in Tehran, Iran completed the SF-12. Reliability was estimated using internal consistency and validity was assessed using known groups comparison and convergent validity. In addition, the factor structure of the questionnaire was extracted by performing both exploratory factor analysis (EFA) and confirmatory factor analysis (CFA).

Results:

In all, 5587 individuals were studied (2721 male and 2866 female). The mean age and formal education of the respondents were 35.1 (SD = 15.4) and 10.2 (SD = 4.4) years respectively. The results showed satisfactory internal consistency for both summary measures, that are the Physical Component Summary (PCS) and the Mental Component Summary (MCS); Cronbach's α for PCS-12 and MCS-12 was 0.73 and 0.72, respectively. Known-groups comparison showed that the SF-12 discriminated well between men and women and those who differed in age and educational status (P < 0.001). In addition, correlations between the SF-12 scales and single items showed that the physical functioning, role physical, bodily pain and general health subscales correlated higher with the PCS-12 score, while the vitality, social functioning, role emotional and mental health subscales more correlated with the MCS-12 score lending support to its good convergent validity. Finally the principal component analysis indicated a two-factor structure (physical and mental health) that jointly accounted for 57.8% of the variance. The confirmatory factory analysis also indicated a good fit to the data for the two-latent structure (physical and mental health).

**Conclusion:**

In general the findings suggest that the SF-12 is a reliable and valid measure of health related quality of life among Iranian population. However, further studies are needed to establish stronger psychometric properties for this alternative form of the SF-36 Health Survey in Iran.

## Background

The Short Form Health Survey SF-36 is a well-known generic health-related quality of life measure that has widely been used worldwide [1-6]. The SF-36 was first presented in a 'developmental' form in 1988 and in 'standard' form in 1990. The standard form reflected improvements in item wording, format and scoring [7]. The SF-36 includes multi-item scales measuring eight health concepts: physical functioning (PF), role limitations due to physical health (RP), bodily pain, general health perception (GH), social functioning (SF), role limitations due to emotional problems (RE), vitality (VT), and mental health (MH). These eight scales are hypothesized to form two distinct clusters related to physical and mental health known as Physical Component Summary (PCS) and Mental Component Summary (MCS). As Gandek et al. explained development of two summary measures from the SF-36 suggested that it might be possible to develop a shorter questionnaire that would produce the SF-36 physical and mental health summary with fewer items [8]. Thus the SF-12 Health Survey was developed as a shorter practical form of the questionnaire to permit its application in large health studies with focus on overall physical and mental health outcomes. Cross-cultural validation studies have shown that there were substantial correlation between the summary measures of the SF-36 and the SF-12 Health Survey [8,9].

Studies using the 12-item Short Form Health Survey (SF-12) have verified the questionnaire as a valid and reliable measure for evaluating overall community health status [10,11] as well as minority ethnic groups [12,13]. The instrument also showed that would properly distinguish a variety of health status among elderly people [14,15]. Similarly well-documented research works have shown that the SF-12 summary scores are acceptable measures of health-related quality of life in patients with different diagnosis such as mental health disorders [16], low back pain [17], retinal diseases [18], osteoarthritis [19] and obesity [20]. However, studies have found that although the instrument showed acceptable validity and reliability, its factor structure in some countries with different cultures might not follow the intended structure of the instrument [15,21].

As a recent publication noted since the ability to accurately and efficiently measure physical and mental health is of great importance in academic and clinical settings and the SF-12 takes less than two minutes to administer and provide such information; the questionnaire is quickly becoming one of the most popular instruments not only in its original country but also among investigators from other nations [22]. The SF-12 is available in many languages such as Spanish, French, German, Swedish, Japanese [9], Italian, Russian, Greek and Chinese [23-26]. However, since the Iranian version of the SF-12 was not available, this study aimed to provide evidence for the psychometric properties of the Iranian version of SF-12 among a general Iranian population. It was hoped this might contribute to the exiting literature and help both researchers and health professionals to have an opportunity to use the questionnaire in their potential research and practice in the future.

## Methods

### The questionnaire and scoring

Permission was asked from the QualityMetric Inc. to develop the Iranian version of SF-12 (License agreement #F1-072706-27488). Since previously we have developed the Iranian version of the SF-36 [27], the SF-12 was extracted from the SF-36 and used in this study. The 12-item Short Form Health Survey (SF-12) is a shorter alternative of the SF-36 instrument that includes 12 questions and 8 scales: physical functioning (PF-2 items on limitations doing moderate activities and climbing several flights of stairs), role limitations due to physical problems (RP-2 items on less accomplishment than one would like to achieve and limitation in kind of work or other activities), bodily pain (BP-1 item on pain interference with one's normal work), general health (GH-1 item on general health perception), vitality (VT-1 item on having energy), social functioning (SF-1 item on interference of physical health or emotional problems with one's social activities), role limitations due to emotional problems (RE-2 items on less accomplishment than one would like to achieve and not being careful in doing activities as usual) and perceived mental health (MH-2 items on feeling calm or peaceful and feeling sad or blue). Response categories for items vary from 2- to 6-point scales and raw scores for items are ranging from 1 to 6. After recoding raw scores for some items (that are BP, GH, VT, and one item from MH); then the raw scores could be transformed in order to provide eight scale scores each ranging from 0 (the worst) to 100 (the best). This method of scoring (summated ratings) assumes that item or items belonging to each scale can be transformed or summed without standardization of scores or item weighting [6,9,28]. We used this method to calculate scale scores. However to calculate the PCS-12 and the MCS-12 scores we used the QualityMetric Health Outcomes Scoring Software 2. The software uses all the 12 items to produce scores for the PCS-12 and the MCS-12 and applies a norm-based scoring algorithm empirically derived from the data of a US general population survey [29]. It has been recommended that the US-derived summary scores, that assume a mean of 50 and a standard deviation (SD) of 10, be used in order to facilitate cross-cultural comparison of results [8,25].

### Data collection

A cross-sectional population-based study was conducted in Tehran, Iran in 2007. The ethics committee of the Iranian Center for Education, Culture and Research (ACECR) approved the study. The Iranian version of SF-12 questionnaire was administered to a random sample of individuals aged 15 years and over. To select a representative sample of the general population a stratified multi-stage area sampling procedure was applied. Every household within 22 municipal districts in Tehran had the same probability to be sampled. A team of trained interviewers collected data and all participants were interviewed in their home. The interviews were carried out with individuals' informed consent.

### Statistical analysis

In addition to descriptive statistics (including floor and ceiling effects), according to International Quality of Life Assessment (IQOLA) Project to assess the psychometric properties of the Iranian version of SF-12 several tests were performed. To test reliability, the internal consistency for summary measures was estimated using Cronbachs' alpha coefficient and alpha equal to or greater than 0.70 was considered satisfactory [30]. Validity was assessed using known groups comparison to test how well the questionnaire discriminates between subgroups of the study sample that differed in gender, age, and educational status. It was expected that women, old people, and those with lower educational levels would have lower scores than men, young people and better educated respondents in all measures. Test for trends was used for comparisons. Furthermore convergent validity was assessed performing item-scale correlations corrected for overlaps. Correlations were calculated using Spearman's correlation coefficient (rho). It was expected that item scores would correlate higher with own hypothesized scale than other scales and PF, RP, BP and GH scores would correlate higher with the PCS-12 whereas the VT, SF, RE and MH scores would correlate higher with the MCS-12. Correlation values of 0.40 or above were considered satisfactory (r ≥ 0.81-1.0 as excellent, 0.61-0.80 very good, 0.41-0.60 good, 0.21-0.40 fair, and 0-0.20 poor) [30].

The factor structure of the questionnaire was extracted by performing both exploratory factor analysis (EFA) and confirmatory factor analysis (CFA). Exploratory factor analysis was performed using the principal component analysis with varimax rotation. It was hypothesized that a two-factor solution would be obtained with eigenvalues greater than 1. Finally, confirmatory factor analysis was performed while a two-factor model (physical component summary and mental component summary) was specified for the analysis. There are varying suggestions in the literature about the number, type and cut-off values for goodness-of-fit required to be reported for confirmatory factor analysis. Accordingly, we report several goodness-of-fit indicators including: goodness of fit index (GFI), adjusted goodness of fit index (AGFI), the root mean square error of approximation (RMSEA), normed fit index (NFI), and comparative fit index (CFI). The GFI and AGFI are chi-square based calculations independent of degrees of freedom. The recommended cut-off values for acceptable values are ≥ 0.90. The RMSEA tests the fit of the model to the covariance matrix. As a guideline, values of < 0.05 indicate a close fit and values below 0.11 are an acceptable fit. The NFI and CFI values range from 0 to 1 with a value of greater than 0.90 being acceptable fit to the data. [31,32].

## Results

In all, 6228 individuals were approached. Of these, 5587 individuals (2721 male and 2866 female) were agreed to take part in the study, giving a response rate of 89.7%. The mean age and formal education of the respondents was 35.1 (SD = 15.4) and 10.2 (SD = 4.4) years respectively. The demographic characteristics of the study sample are shown in Table 1.

**Table 1:** Demographic characteristics of the study sample (n = 5587)

		**Number (%)**
**Age groups (year)**		

	15-24	1843 (33.0)

	25-44	2253 (40.3)

	45-64	1212 (21.7)

	≥ 65	279 (5.0)

	Mean (SD)	35.1 (15.4)

**Gender**		

	Male	2721 (48.7)

	Female	2866 (51.3)

**Marital status**		

	Single	2051 (36.7)

	Married	3342 (59.8)

	Widowed/divorced	194 (3.5)

**Educational status**		

	Primary	978 (17.5)

	Secondary	3430 (61.4)

	Higher	1179 (21.1)

	Mean year (SD)	10.2 (4.4)

**Employment status**		

	Employed	1985 (35.5)

	Housewife	1715 (30.7)

	Student	1036 (18.5)

	Unemployed	496 (8.9)

	Retired	355 (6.4)

Table 2 shows the descriptive statistics for the SF-12 scales. Both summary measures exceeded the 0.70 level indicating satisfactory results (Cronbach's α for PCS-12 and MCS-12 was 0.73 and 0.72, respectively). The mean score for the PCS-12 was 50.1 (SD = 8.5) and for the MCS-12 it was 46.3 (SD = 10.4). For both the PCS-12 and the MCS-12 the percentage of respondents scoring at the lowest level (i.e. floor effect) and at highest level (i.e. ceiling effect) was almost nothing (frequency was 1 for each).

**Table 2:** Item description and descriptive statistics for the SF-12 component summary scores (n = 5587)*

**SF-12 items (scale)**	**Mean row scores (SD)**	**95% CI**	**Response frequencies (%)**					
			**1**	**2**	**3**	**4**	**5**	**6**

Limitations in moderate physical activities (PF)	2.66 (0.60)	2.65-2.68	7.0	19.6	73.4	-	-	-

Limitations in climbing several flights of stairs (PF)	2.65 (0.64)	2.63-2.67	9.7	15.7	74.6	-	-	-

Accomplished less due to physical health (RP)	1.70 (0.20)	1.69-1.72	29.6	70.4	-	-	-	-

Limited in kind of work or activities due to physical health (RP)	1.72 (0.44)	1.71-1.74	27.6	72.4	-	-	-	-

Pain interference with work inside or outside home (PB)**	4.29 (0.91)	4.27-4.32	2.0	3.6	8.6	34.5	51.4	-

Health rating in general (GH)**	3.15 (1.10)	3.12-3.18	4.4	26.1	34.4	19.8	15.2	-

Interference of physical health or emotional problems with social activities (SF)	3.92 (1.08)	3.89-3.95	2.8	7.9	22.4	28.5	38.4	-

Accomplished less due to emotional problems (RE)	1.64 (0.23)	1.63-1.66	35.7	64.3	-	-	-	-

Not careful in work or activities due to emotional problems (RE)	1.67 (0.22)	1.66-1.68	32.8	67.2	-	-	-	-

Having a lot of energy (VT)**	4.04 (1.38)	4.00-4.07	4.7	9.4	22.4	19.5	28.4	15.5

Feel calm and peaceful (MH)**	4.33 (1.35)	4.29-4.36	4.1	6.6	15.8	19.5	33.3	20.6

Feel downhearted and blue (MH)	4.39 (1.57)	4.36-4.43	3.2	6.5	7.6	33.9	28.1	20.7

**Summary components**	**PCS-12**	**MCS-12**						

Mean (SD)***	50.1 (8.5)	46.3 (10.4)						

95% CI	49.8-50.3	46.1-46.6						

Cronbach's α	0.73	0.72						

Skewness	-0.98	-0.68						

Minimum (% floor)	14.74 (0.0)	9.17 (0.0)						

Maximum (% ceiling)	68.74 (0.0)	70.35 (0.0)						

* The format adapted from [25].

**Item recoded in order to make all response frequencies in the same direction. Now for all 12 items higher scores indicate better condition.

*** Derived from QualityMetric Health Outcomes Scoring Software 2.

Known groups comparison showed that the SF-12 discriminated well between subgroups of people who were differed in gender, age and educational status. As hypothesized women, older people and respondents with lower education reported poorer health status than men, younger participants and those with a better educational status in all measures (Tables 3).

**Table 3:** Comparison of the SF-12 scores for the general population by gender, age and educational status

	**Physical component summary**	**Mental component summary**
	**Mean (SD)**	**Mean (SD)**

**Gender**		

Male (n = 2721)	50.9 (8.1)	47.3 (10.1)

Female (n = 2866)	49.2 (8.9)	45.3 (10.5)

P value*	< 0.001	< 0.001

**Age groups**		

15-24 (n = 1843)	53.5 (6.3)	48.3 (9.5)

25-44 (n = 2253)	50.8 (7.5)	45.7 (10.6)

45-64 (n = 1212)	45.8 (8.8)	44.8 (10.6)

≥ 65 (n = 279)	38.1 (10.1)	44.4 (10.7)

P value*	< 0.001	< 0.001

**Educational status**		

Primary (n = 978)	45.5 (10.3)	44.3 (10.2)

Secondary (n = 3430)	50.9 (7.9)	46.5 (10.3)

Higher (n = 1179)	51.1 (7.4)	47.4 (10.4)

P value*	< 0.001	< 0.001

* Derived from test for trends.

In addition the results from correlation analysis showed that item scores correlated higher with own hypothesized scale than other scales and that the PF, RP, BP and GH subscales correlated higher with the PCS-12 score, while the VT, SF, RE and MH subscales more correlated with the MCS-12 score lending support to its good convergent validity. (Table 4).

**Table 4:** Item-scale correlation matrix for the eight SF-12 scales and summary measures*

	**PF**	**RP**	**BP**	**GH**	**SF**	**RE**	**VT**	**MH**	**PCS**	**MCS**
*PF*										

PF1	**0.84**	0.42	0.25	0.37	0.31	0.25	0.26	0.23	**0.62**	0.17

PF2	**0.85**	0.44	0.24	0.36	0.32	0.26	0.29	0.24	**0.61**	0.20

*RP*										

RP1	0.42	**0.89**	0.27	0.34	0.36	0.43	0.25	0.24	**0.62**	0.30

RP2	0.44	**0.87**	0.29	0.34	0.38	0.40	0.24	0.25	**0.65**	0.29

*BP*										

BP1	0.28	0.32	**1.00**	0.27	0.34	0.25	0.22	0.22	**0.58**	0.23

*GH*										

GH1	0.42	0.38	0.27	**1.00**	0.36	0.30	0.35	0.31	**0.61**	0.35

*SF*										

SF1	0.36	0.41	0.34	0.36	**1.00**	0.43	0.35	0.45	0.36	**0.66**

*RE*										

RE1	0.25	0.41	0.21	0.27	0.37	**0.91**	0.25	0.33	0.12	**0.70**

RE2	0.29	0.42	0.24	0.28	0.40	**0.89**	0.26	0.34	0.17	**0.67**

*VT*										

VT1	0.32	0.28	0.22	0.35	0.35	0.28	**1.00**	0.48	0.35	**0.53**

*MH*										

MH1	0.24	0.23	0.18	0.27	0.34	0.27	0.49	**0.82**	0.15	**0.61**

MH2	0.19	0.21	0.16	0.25	0.40	0.34	0.28	**0.78**	0.05	**0.68**

* Figures are Spearman's correlation coefficient (rho). All correlations were significant at the 0.01 levels. Correlation values of 0.40 or above were considered satisfactory (correlations ≥ 0.81-1.0 as excellent, 0.61-0.80 very good, 0.41-0.60 good, 0.21-0.40 fair, and 0-0.20 poor) [30].

Principal component analysis with varimax rotation loaded two factors. The results are shown in Table 5. Eigenvalues for the two factors that explained most of the variance observed was 4.52 and 1.27 respectively. The two-factor structure (physical and mental health) jointly accounted for 57.8% of the variance. The results showed that PF, RP, BP and GH items loaded higher on the physical component and VT, SF, RE and MH loaded higher on the mental component.

**Table 5:** Factor structure of the SF-12 derived from principal component analysis*

	**Factor 1**	**Factor 2**
**Physical functioning (PF)**		

Limitations in moderate physical activities (PF1)	**0.75**	0.07

Limitations in climbing several flights of stairs (PF2)	**0.74**	0.10

**Role physical (RP)**		

Accomplished less due to physical health (RP1)	**0.67**	0.26

Limited in kind of work or activities due to physical health (RP2)	**0.72**	0.23

**Bodily pain (BP)**		

Pain interference with work inside or outside home (PB1)	**0.48**	0.30

**General health (GH)**		

Health rating in general (GH1)	**0.51**	0.34

**Social functioning (SF)**		

Interference of physical health or e-motional problems with social activities (SF1)	0.38	**0.60**

**RE**		

Accomplished less due to emotional problems (RE1)	0.27	**0.61**

Not careful in work or activities due to emotional problems (RE2)	0.32	**0.60**

**Vitality (VT)**		

Having a lot of energy (VT1)	0.22	**0.61**

**MH**		

Feel calm and peaceful (MH1)	0.07	**0.70**

Feel down hearted and blue (MH2)	0.05	**0.71**

**Eigenvalues**	4.52	1.27

**Variance explained (%)**	37.7	20.1

* Values equal or greater than 0.4 were considered satisfactory.

**Figure 1:**
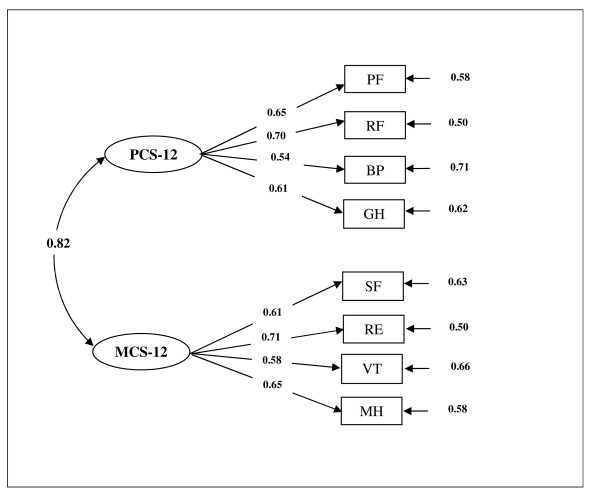
**A two-factor model for the SF-12 obtained from confirmatory factor analysis**.

Finally, the results for confirmatory factor analysis are shown in Figure 1. The two-factor model, that is physical component summary (PCS-12) and mental component summary (MCS-12), was specified and tested. The results provided a good fit to the data lending support to the original hypothesized structure of the questionnaire with GFI = 0.96, AGFI = 0.93, RMSE = 0.090, 95% CI RMSE = 0.085 to 0.095, NFI = 0.93, and CFI = 0.93.

## Discussion

This is the first study that reports on psychometric properties of the Iranian version of SF-12 among a general Iranian population. The results showed that the instrument is a reliable and valid measure that can be used in monitoring and measuring of population health status. Since the present study used the norm-based scoring algorithms for calculating the PCS-12 and MCS-12 scores, the results from this study also can be used for cross-cultural quality of life comparisons. Similarly the validity of the SF-12 in different cultures is well documented. For instance, the findings from an Italian study showed that the instrument has good validity and could be applied both among Italian general population and specific patients groups [23]. Psychometric properties of the Greek version of SF-12 also provided evidence on the validity of the instrument and supported its use in Greek health-related quality of life studies [25].

The Iranian version of the SF-12 was extracted from the Iranian version of the SF-36. The translation of the SF-36 in Iran went through a rigorous method and was approved by the questionnaire developers. Thus similar to our previous study we did not counter any difficulties in data collection. The questionnaire received well and it was acceptable to almost all participants [for details see [27]]. In addition, Face-to-face administration of the questionnaire allowed the interviewers to collect data without any missing data. Some self-administered applications of the SF-12 have found very high incompletion rates [33]. However, one should note that the findings from this study might not hold when the instrument is used in a self-administered mode.

This study used a relatively large sample of the general population. Therefore as it has been suggested [34] the result of this study might be considered as Iranian normative data for the 12-item Short Form Health Survey (SF-12) and perhaps could be used as a basis for comparison with specific populations in the future studies. However one might argue that a sample from the urban capital is not necessarily representative of the entire country. In general this is true but since Tehran has become a multicultural metropolitan area it has been suggested that a sample from the general population in Tehran at least could be regarded as a representative sample of urban population in Iran [27].

The mean score for the PCS-12 and MCS-12 was 50.1 and 46.3 respectively (Table 2). Compared to the results from nine countries, this study showed lower scores for the PCS-12 and the MCS-12 among a general Iranian population [8]. The findings also indicated that mental health related quality of life among Iranian population was lower than the physical health related quality of life. In addition, no floor or ceiling effects were observed for the SF-12 summary scores in this population-based study showing that these summary scores are useful indicators of people's health status (Table 2). Similar result also was reported from Greece [25].

Known-groups comparison indicated that the SF-12 summary scores were able to distinguish very well between subgroups of the respondents who differed in gender, age and educational status. The study findings showed that women, old age people and people with lower educational status had poorer health compared to men, the younger respondents and those with better educational status. These are consistent with results from other studies carried out in different countries such as Italy and Greece [23,25]. It seems that the SF-12 summary scores, similar to the SF-36 scores, are highly dependent on gender, age and education [e.g. [35]].

The hypothesis regarding the item component correlations also showed desirable results. As expected the PF, RP, BP and GH subscales correlated higher with the PCS-12 score while the VT, SF, RE and MH more correlated with the MCS-12 score (Table 4). This finding is somewhat different from those that were reported by the Ware et al. where physical functioning, role physical and bodily pain correlated most highly with the PCS and mental health, role emotional, and social functioning correlated most highly with MCS; and vitality, general health and social functioning had a relatively high correlation with both components [9]. However, a number of studies have shown that vitality item has appeared to correlate higher with the PCS than with the MCS score [25]. It is argued this might be due to cultural differences among people from different countries or simply this might be occurred due to translation problems [27,36]. In addition, it has been reported that even translation of concepts such as social functioning could be difficult in some Asian cultures [e.g. see [6]]. As Ware indicates the most important empirical point that should be noted is the fact that scales that load highest on the physical component are most responsive to treatment that change physical morbidity whereas scales loading highest on the mental component respond to drugs and therapies that target mental health [37].

In general, the psychometric tests of the Iranian version of SF-12 showed satisfactory results. Both principal component analysis with varimax rotation and confirmatory factor analysis supported a two-factor structure for the instrument that ensured the original conceptual model of the instrument [8,9]. However a recent publication suggested that using correlated oblique model would also provide reliable information for the SF-12 summary scores [38].

Although this study did not provide evidence for test-retest reliability, responsiveness to change or other psychometric tests; the findings showed that the Iranian version of SF-12 is a reliable measure for measuring health-related quality of life. The future studies could focus on other psychometric properties of the questionnaire and also on different applications of the questionnaire as a recent study has suggested even it is a useful index in order to evaluate cost-effectiveness of healthcare interventions [39].

## Conclusion

In general the findings suggest that the SF-12 is a reliable and valid measure of health related quality of life among Iranian population. However, further studies are needed to establish stronger psychometric properties for this alternative form of the SF-36 Health Survey in Iran.

## Abbreviations

SF-12: The 12-item Short Form Health Survey; PF: Physical Functioning; RP: Role Physical; BP: Bodily Pain; GH: General Health; VT: Vitality; SF: Social Functioning; RE: Role Emotional; MH: Mental Health; IQOLA: International Quality of Life Assessment; PCS: Physical Component Summary; MCS: Mental Component Summary.

## Competing interests

The authors declare that they have no competing interests.

## Authors' contributions

AM was the main investigator, provided the questionnaire, carried out the analysis, and wrote the paper. MV contributed to all aspects of the study including the analysis and the writing process. SJM contributed to the study design, and analysis. SO contributed to the study design and the project management. All authors read and approved the manuscript.

## Pre-publication history

The pre-publication history for this paper can be accessed here:


